# Harnessing Semi-Supervised Machine Learning to Automatically Predict Bioactivities of Per- and Polyfluoroalkyl Substances (PFASs)

**DOI:** 10.1021/acs.estlett.2c00530

**Published:** 2022-08-26

**Authors:** Hyuna Kwon, Zulfikhar A. Ali, Bryan M. Wong

**Affiliations:** † Department of Chemical & Environmental Engineering, University of California-Riverside, Riverside, California 92521, United States; ‡ Department of Physics & Astronomy, University of California-Riverside, Riverside, California 92521, United States

**Keywords:** per- and polyfluoroalkyl substances, PFAS, machine learning, bioactivity, semi-supervised learning

## Abstract

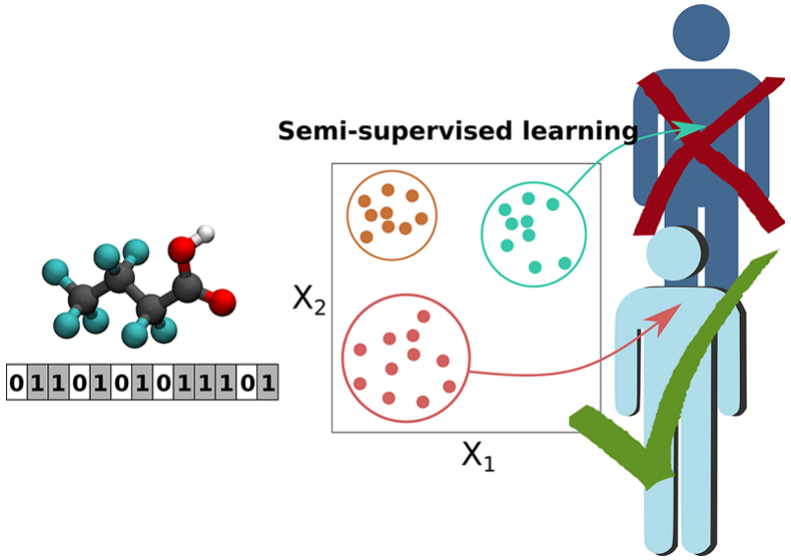

Many per- and polyfluoroalkyl substances (PFASs) pose significant health hazards due to their bioactive and persistent bioaccumulative properties. However, assessing the bioactivities of PFASs is both time-consuming and costly due to the sheer number and expense of *in vivo* and *in vitro* biological experiments. To this end, we harnessed new unsupervised/semi-supervised machine learning models to automatically predict bioactivities of PFASs in various human biological targets, including enzymes, genes, proteins, and cell lines. Our semi-supervised metric learning models were used to predict the bioactivity of PFASs found in the recent Organisation of Economic Co-operation and Development (OECD) report list, which contains 4730 PFASs used in a broad range of industries and consumers. Our work provides the first semi-supervised machine learning study of structure–activity relationships for predicting possible bioactivities in a variety of PFAS species.

## Introduction

Since the 1930s,^1^ per- and polyfluoroalkyl substances (PFASs) have been used in several consumer products (including fire-fighting foams) due to their outstanding stability and water/oil repellant properties.^2^ However, these compounds pose significant risks to the environment and biosystems. The presence of PFASs in surface water and groundwater can result in exposure to organisms, subsequently leading to accumulation in the body, with adverse effects on the liver, kidneys, blood, and immune system.^2,3^ Because of these deleterious effects, there is a pressing need to identify and understand the bioactivity of PFAS-based compounds that can adversely affect human health.

For these reasons, several international groups including the Organisation of Economic Co-operation and Development (OECD), United States Environmental Protection Agency, Food and Drug Administration, European Chemicals Agency, European Food Safety Authority, and Ministry of Ecology and Environment (China) continue to monitor PFASs that are produced in the global market.^4,5^ According to a 2018 OECD report, more than 4700 PFASs currently exist as manufacturers bring new forms of PFASs into industrial and consumer products (it is worth pointing out, however, that not all 4700 structures exist in commerce). Nevertheless, among the wide varieties of PFAS molecules, the potential hazards of these new forms remain largely unknown.

Due to the sheer number of PFAS species, *in vivo* and *in vitro* biological experiments are both time-consuming and costly. As such, the construction of predictive and reliable quantitative-structure activity relationship (QSAR) models^6−8^ is essential for assessing the bioactivities of these contaminants (even for PFAS species that are yet to be made). Specifically, a QSAR model that can accurately predict the bioactivities of PFASs can be harnessed to screen several of these contaminants, saving immense time and experimental resources. While there have been prior machine learning studies on PFAS molecules,^9,10^ most of these approaches used supervised learning techniques to suggest *general* structure–bioactivity trends after postprocessing of the data (i.e., the focus was on aggregate data for all targets as opposed to analyzing chemical trends specific to each target).

In this work, we present a new QSAR model using semi-supervised metric learning techniques to assess which chemical functional groups affect bioactivities toward specific biological targets. Semi-supervised learning is a different machine learning approach that has the advantages of both supervised and unsupervised learning. It can be used on a dataset with primarily unlabeled data and only a few labeled data. Like unsupervised learning, it can also automatically cluster unlabeled data. Our approach is integrated with molecular docking calculations to predict possible bioactivities of PFAS molecules based on their chemical functional groups and specific biological targets (e.g., genes, proteins, or cell lines). Our approach first combines dimension reduction methods with clustering methods to classify PFASs based on their molecular structures. We then apply a semi-supervised metric learning method to improve classification accuracy. Finally, we use a molecular docking approach to shed light on the physicochemical reasons for their bioactivity. Our study provides the first unsupervised/semi-supervised learning approach for screening potentially bioactive PFAS molecules beyond conventional supervised learning or QSAR approaches.

## Methods

**Figure 1:**
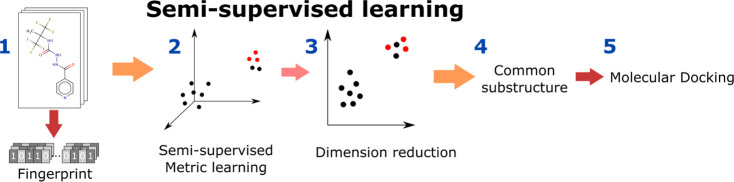
Machine-learning-based workflow for QSAR construction to predict bioactivity of PFASs.

Our QSAR machine-learning framework, shown in Figure 1, utilizes four sequential steps followed by a reasoning/validation step: (1) collecting a training dataset from verified open-source databases, (2) encoding those compounds into molecular fingerprints, (3) clustering the data to predict chemical properties based on the molecular fingerprints and assessing the performance of the models, (4) evaluating the clustering by choosing the optimal model and predicting molecular groups responsible for bioactivity based on the clustering, and (5) molecular docking simulations to rationalize the role of the chemical functional groups. All of our machine learning algorithms are publicly available (see Supporting Information).

In our first step, we obtained datasets from comprehensive open-source databases, including PubChem’s BioAssay,^11^ Maximum Unbiased Validation,^12^ Toxicology in the 21st Century,^13^ beta-secretase 1,^14^ and blood-brain barrier penetration datasets,^15^ which are available from the Supporting Information of ref (10). We used two different datasets without further modification from ref (10): (1) the CF dataset, which includes substances containing at least one −CF– moiety (62 043 molecules), and (2) the C3F6 dataset, which includes substances containing a perfluoroalkyl moiety with three or more carbons (1012 molecules). For both datasets, we used bioactivity data against 26 biological targets.

Encoding the compounds to molecular fingerprints followed next in our framework. We used the extended connectivity fingerprint (ECFP) featurization^16^ with a default diameter of 4 (i.e., ECFP4), which considers a maximum of four neighbors. ECFPs are topological molecular representations developed for substructure and similarity searching. By encoding molecular structures into fingerprints, we obtained a binary array with a constant length of 2048, making it a convenient input for the unsupervised/semi-supervised learning models. Furthermore, since the simplified molecular-input line-entry system (SMILES) sequences for all PFAS molecules are readily available, they can be easily converted into fingerprint-based representations using the RDKit software package.^17^

We then applied semi-supervised metric learning to the generated fingerprints by training machine learning models to predict the bioactivities of PFAS molecules by first (a) *reducing the dimension of the fingerprint datasets* and then (b) *classifying/clustering them* (see Figure 1). Our QSAR model used a semi-supervised metric learning algorithm to automatically group/classify molecules with similar bioactivities. Metric learning has two main advantages: (1) its predictions are more efficient/accurate since the model distinctly separates new molecular representations according to their bioactivities (by reducing the distance metric between the same-labeled pair of data and increasing the distance between opposite-labeled pair of data), and (2) it automatically generates a vector-shaped representation from the molecular fingerprint and can be directly integrated with conventional dimension reduction methods. The final clusters were selected based on the best Silhouette score, which analyzes the distances of each data point to its cluster and neighboring clusters.^18^ In short, a higher Silhouette score indicates more distinct and separated clusters. We then identified which substructures or molecular functional groups played essential roles in determining the bioactivity of the molecules.

Lastly, we conducted several molecular docking calculations using Autodock^19^ to elucidate the physicochemical reasons for the bioactivity trends obtained from our QSAR model (i.e., using ligand-protein binding conformations to rationalize the role of chemical substructures that induces bioactivity on biological targets.)

## Results and Discussion

### 3.1 Unsupervised vs Semi-supervised Machine Learning

To systematically evaluate the performance of our semi-supervised metric approach, we first performed traditional unsupervised machine learning and compared the performance of the two models. To maintain a concise discussion of our results, the Supporting Information contains a detailed analysis and comparison of our unsupervised vs semi-supervised machine learning results. Figure S1 shows our clustering results using unsupervised machine learning on the C3F6 dataset, and Figure S2 shows a comparison between the unsupervised and semi-supervised results using the CF dataset on two different targets. Table S3 summarizes the substructures that induce bioactivity as predicted from our unsupervised learning calculations. In summary, our extensive analyses in the Supporting Information show that semi-supervised metric learning performed significantly better than unsupervised machine learning; as such, we only focus on the results of the former in this manuscript.

### 3.2 Semi-Supervised Metric Learning

**Figure 2:**
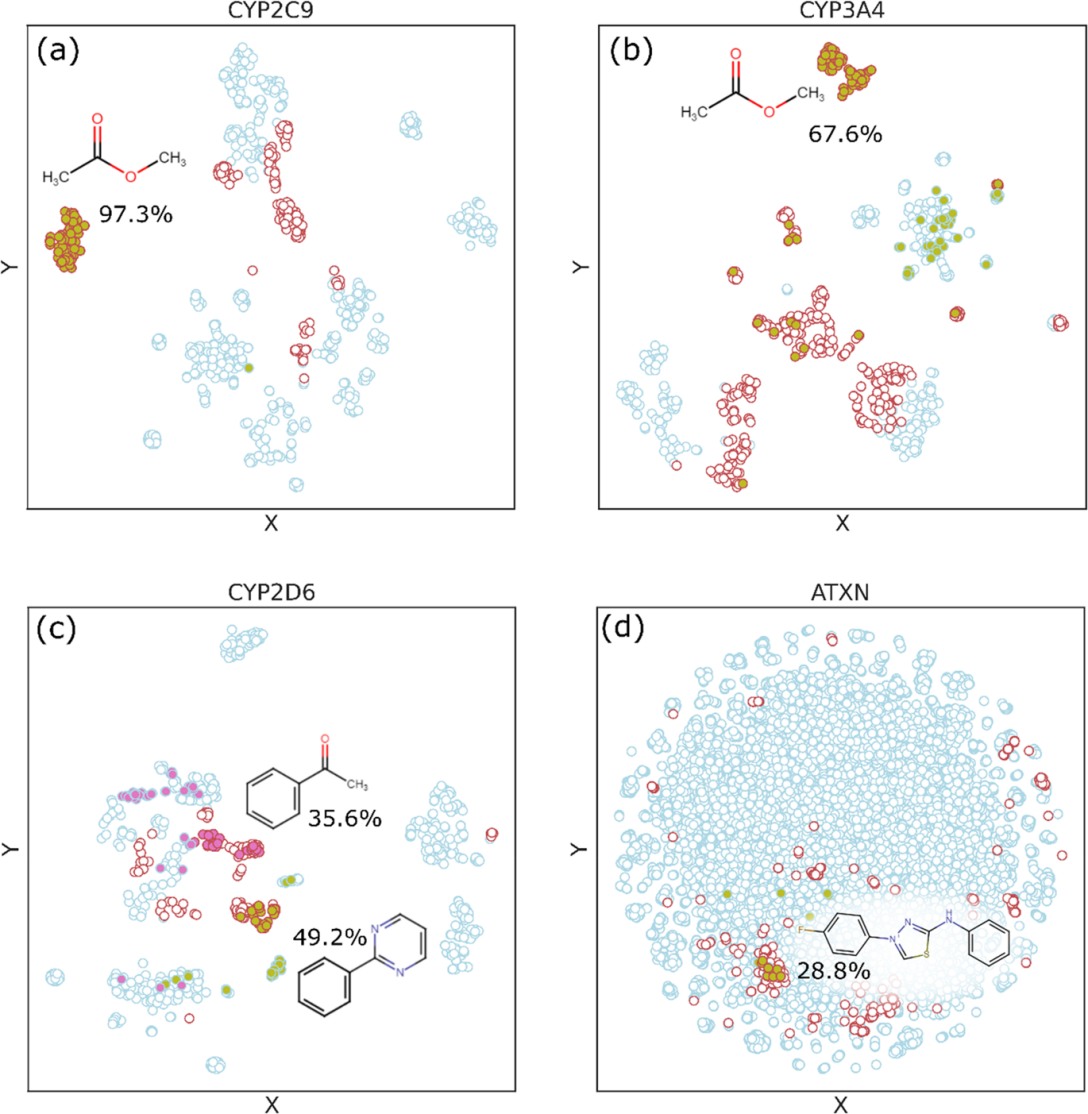
Distribution of molecules in the CF dataset using semi-supervised metric learning. Each point represents a molecule that is either bioactive (red circular edges) or inactive (light blue circular edges) toward (a) CYP2C9, (b) CYP3A4, (c) CYP2D6, and (d) ATXN. The olive green-filled circles represent molecules having the substructure depicted in the plot; i.e., (a, b) ester groups, (c) phenylprimidyl groups, and (d) 4-benzyl-2-(4-fluorophenyl)-1,2-thiazole. The pink-filled circles in (c) represent molecules with phenylethanone. The percentage value represents the ratio of the number of bioactive molecules within the identified substructure. Table S3 lists the predicted substructures for specific targets.

Figure 2 displays true-positive ratios and classifications between bioactive/inactive molecules on four representative targets that show the best performance in the CF dataset using semi-supervised metric learning (for example, in Figure 2a, we obtain a true-positive ratio of 97.3% by computing the following ). Using the Maximum Common Structure (MCS) module in the RDKit software package on bioactive molecules, we found that the ester functional group is the critical substructure that causes bioactivity on Cyps (Figure 2a–c) and ATXN (Figure 2d). Table S4 summarizes the substructures predicted to play a vital role in bioactivity toward nine different targets. The other 17 targets did not demonstrate as distinct clustering as the nine targets in Table S4 due to a relatively weak correlation between molecular structure and bioactivity.

We used structural alerts to cross-check the validity of the predicted substructures that play a crucial role in bioactivity. Within the bioinformatics community, structural alerts are molecular functional groups associated with a particularly adverse outcome, in our case, bioactivity.^20,21^ We cross-referenced the CheMBL dataset to our machine learning results since it contains structural alert information for some PFAS molecules.^22^Figure S3 shows structural alerts of the molecules that are bioactive on CYP2C9, and, as mentioned previously, the ester group was found to be the critical structure that induces interaction with Cyps.^23,24^

### 3.3 Interactions between PFASs and Targets

We carried out molecular docking calculations with Autodock^21^ to rationalize the underlying molecular causes of bioactivities in PFASs and predict their interaction with target enzymes. The Supporting Information gives additional details of our molecular docking calculations. We successfully docked all PFASs into the active sites of the targets and binned the binding affinity results based on their bioactivity with the target. Figure S5 displays one of the bioactive structures with the ester group of the CYP2C9-PFAS complex, methyl 4-[2-propyl-1-({[4-trifluoromethyl)phenyl]sulfonyl}amino)-2-hexen-1-yl]benzoate.

**Figure 3:**
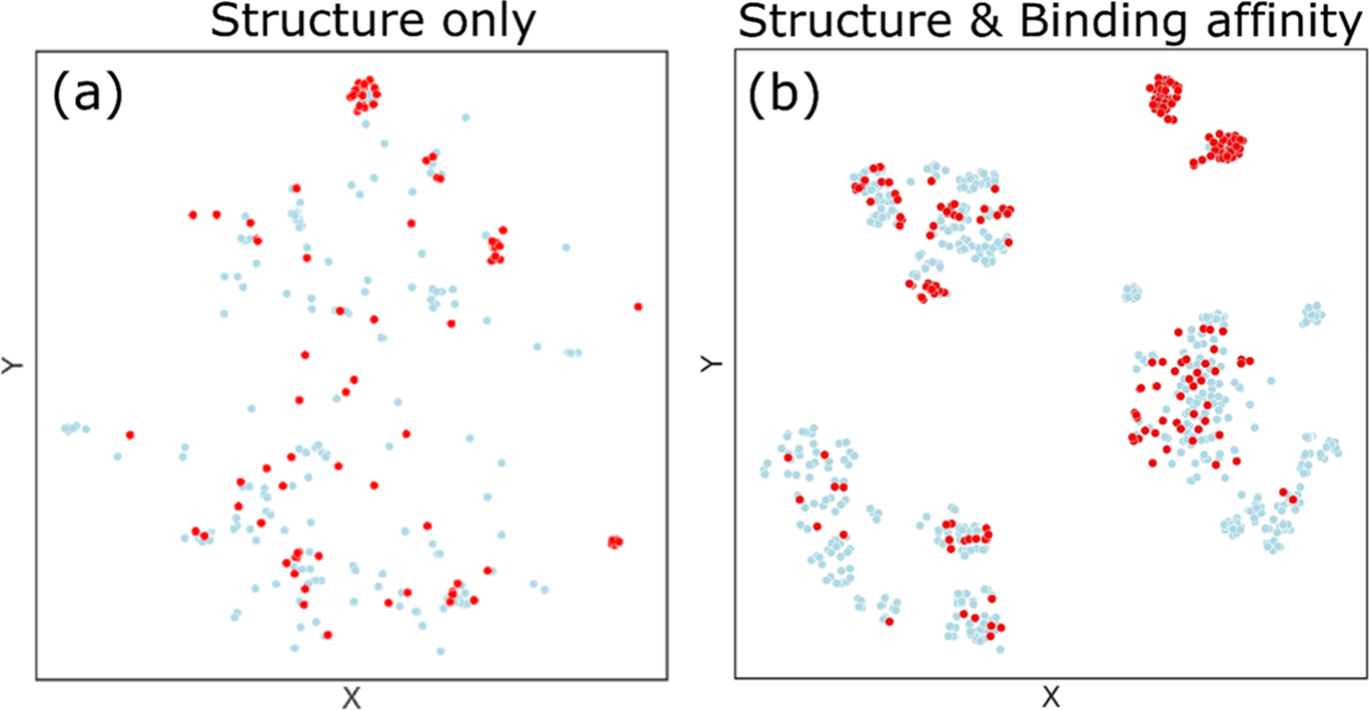
Clustering of molecules predicted with unsupervised learning (dimension reduction) on CF datasets containing (a) chemical structures and (b) chemical structures and binding affinities with CYP2C9. Each point represents a molecule that is either bioactive (red) or inactive (blue) toward CYP2C9.

To verify the correlation between the Autodock binding affinities and their bioactivity, we performed a dimension reduction procedure using unsupervised learning on the CF dataset, which consists of molecular structures with binding affinity data (see Figure 3). We used unsupervised learning here to make the point that unsupervised learning underperforms when only structural data is provided. Specifically, if the classification accuracy is improved with additional feature inputs, those features must contain some information to discriminate among the population.^25,26^ In other words, if the inclusion of binding affinity data enhances the clustering accuracy, it provides another codescriptor for bioactivity. Indeed, Figure 3 shows that descriptors consisting of chemical structures *and* binding affinity data (panel b) give a better separation/distinction between active and inactive molecules compared to the unsupervised learning results based only on chemical structures (panel a).

### 3.4 Bioactivity Predictions on the OECD Dataset

In 2018, the Global Perfluorinated Chemicals Group^27^ within the OECD published a list of 4730 PFASs to develop regulatory approaches for reducing the use of perfluorinated substances in products. However, researchers have yet to discover the bioactivities of the molecules in the list. Using the QSAR model developed in this work, we give predictions and a rationale for the bioactivities of molecules in the OECD list.

We performed molecular docking calculations on molecules containing the ester group among the OECD list to verify similar binding conformations. Of the 4730 PFASs in the OECD list, 414 have an ester functional group. Figure S6 shows four different representative ester-containing molecules bound to CYP2C9. In particular, the ester-containing molecules in the OECD list bind strongly with Fe^2+^ of the HEME group (an active site of Cyp enzyme), which is similar to the binding interactions that we observed in the CF dataset. Therefore, we expect a large portion of the 414 ester-containing molecules among the OECD list to form strong bonds with Fe^2+^ of the HEME group with a similar conformation, leading to bioactivity toward Cyp enzymes. Furthermore, based on our docking calculations, 87.7% of these 414 molecules have a stronger binding affinity than −5 kcal/mol (the average binding affinity is −5.77 kcal/mol), which falls in the range of the mean binding affinity of the bioactive molecules from the CF dataset.

**Figure 4:**
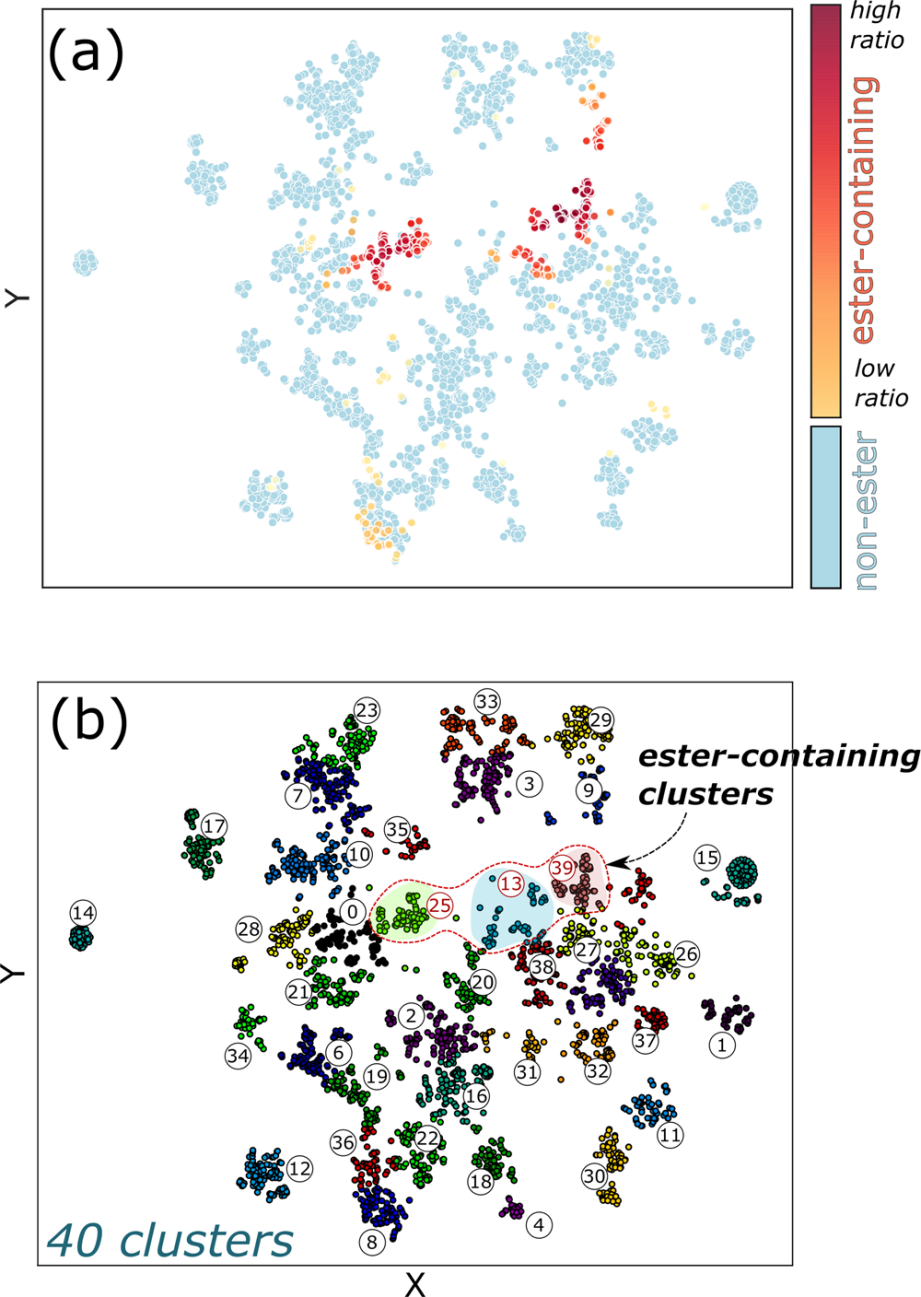
(a) OECD dataset classified by PC t-SNE and clustered based on the k-means clustering method. The orange and yellow dots represent ester-containing molecules. The colors closer to red (yellow) represent a higher (lower) concentration of bioactive molecules. (b) PFAS molecules included in the OECD list are grouped into 40 clusters. Each point represents a molecule, and clusters 13, 25, and 39 denote a high ratio of ester-containing groups.

We then clustered the OECD dataset into 40 clusters using the k-means clustering method. Using both the clustered results (Figure 4b) and the distribution of ester-group-containing molecules (Figure 4a), we found that clusters 13, 25, and 39 contain ester functional groups. Analyzing the CF dataset, we found that the ester group plays a possible role in bioactivity toward Cyp enzymes; that is, molecules in these clusters have a high probability of being bioactive against CYP2C9 and CYP3A4.

In summary, we have developed a new QSAR model validated with CheMLB structural alerts and molecular docking calculations, which constitutes the first application of semi-supervised metric learning for predicting/rationalizing bioactivities in PFASs. Using a semi-supervised metric learning algorithm, our machine-learning-based QSAR model accurately identified specific substructures, such as ester-containing groups, that play a possible role in determining bioactivities. With our semi-supervised learning approach, we obtained a distinct classification between bioactive and inactive molecules, resulting in an accuracy of up to 97.3% in the CF dataset. We also used semi-supervised metric learning to automatically classify/cluster and predict functional groups that could possibly play a role in bioactivity.

In addition, our machine learning model proposed a few significant substructures that could induce bioactivity, which were subsequently examined with molecular docking calculations. Most importantly, our machine learning predictions on bioactivities can provide a more efficient screening of potentially bioactive PFASs that can be used to complement *in vitro* assessments. All of our machine learning algorithms are publicly available (see Supporting Information), and we anticipate that researchers can further extend our methodology to screen other contaminants or analyze the potential bioactivity of PFAS molecules.

## Supporting Information Available

Additional details on unsupervised and semi-supervised metric machine learning methods, additional details on molecular docking calculations, unsupervised machine learning results (PDF)
Open-source Python codes for all the machine learning algorithms used in this work: https://github.com/kha8128/PFAS_ML.git

## References

[ref1] Hepburn E.; Madden C.; Szabo D.; Coggan T. L.; Clarke B.; Currell M. Contamination of Groundwater with Per- and Polyfluoroalkyl Substances (PFAS) from Legacy Landfills in an Urban Re-Development Precinct. Environ. Pollut. 2019, 248, 101–113. 10.1016/j.envpol.2019.02.018. 30784829

[ref2] Blake B. E.; Pinney S. M.; Hines E. P.; Fenton S. E.; Ferguson K. K. Associations between Longitudinal Serum Perfluoroalkyl Substance (PFAS) Levels and Measures of Thyroid Hormone, Kidney Function, and Body Mass Index in the Fernald Community Cohort. Environ. Pollut. 2018, 242, 894–904. 10.1016/j.envpol.2018.07.042. 30373035 PMC6309414

[ref3] Guillette T. C.; McCord J.; Guillette M.; Polera M. E.; Rachels K. T.; Morgeson C.; Kotlarz N.; Knappe D. R. U.; Reading B. J.; Strynar M.; Belcher S. M. Elevated Levels of Per- and Polyfluoroalkyl Substances in Cape Fear River Striped Bass (Morone Saxatilis) Are Associated with Biomarkers of Altered Immune and Liver Function. Environ. Int. 2020, 136, 105358 10.1016/j.envint.2019.105358. 32044175 PMC7064817

[ref4] Organisation for Economic Co-operation and Development (OECD). Toward a New Comprehensive Global Database of Per- and Polyfluoroalkyl Substances (PFASs):. OECD Environment, Health and Safety Publications Series on Risk Management No. 39; OECD: Paris, 2018; pp 1–24. See the following: https://www.oecd.org/officialdocuments/publicdisplaydocumentpdf/?cote=ENV-JM-MONO(2018)7&doclanguage=en.

[ref5] Cousins I. T.; Dewitt J. C.; Glüge J.; Goldenman G.; Herzke D.; Lohmann R.; Miller M.; Ng C. A.; Scheringer M.; Vierke L.; Wang Z. Strategies for Grouping Per-and Polyfluoroalkyl Substances (PFAS) to Protect Human and Environmental Health. Environ. Sci.: Process. Impacts. 2020, 22, 1444–1460. 10.1039/D0EM00147C. 32495786 PMC7585739

[ref6] Hansch C.; Fujita T. P-σ-π Analysis. A Method for the Correlation of Biological Activity and Chemical Structure. J. Am. Chem. Soc. 1964, 86, 1616–1626. 10.1021/ja01062a035.

[ref7] Cherkasov A.; Muratov E. N.; Fourches D.; Varnek A.; Baskin I. I.; Cronin M.; Dearden J.; Gramatica P.; Martin Y. C.; Todeschini R.; Consonni V.; Kuz’min V. E.; Cramer R.; Benigni R.; Yang C.; Rathman J.; Terfloth L.; Gasteiger J.; Richard A.; Tropsha A. QSAR Modeling: Where Have You Been? Where Are You Going To?. J. Med. Chem. 2014, 57, 4977–5010. 10.1021/jm4004285. 24351051 PMC4074254

[ref8] Neves B. J.; Braga R. C.; Melo-Filho C. C.; Moreira-Filho J. T.; Muratov E. N.; Andrade C. H. QSAR-Based Virtual Screening: Advances and Applications in Drug Discovery. Front. Pharmacol. 2018, 9, 1275 10.3389/fphar.2018.01275. 30524275 PMC6262347

[ref9] Raza A.; Bardhan S.; Xu L.; Yamijala S. S. R. K. C.; Lian C.; Kwon H.; Wong B. M. A Machine Learning Approach for Predicting Defluorination of Per- And Polyfluoroalkyl Substances (PFAS) for Their Efficient Treatment and Removal. Environ. Sci. Technol. Lett. 2019, 6, 624–629. 10.1021/acs.estlett.9b00476.

[ref10] Cheng W.; Ng C. A. Using Machine Learning to Classify Bioactivity for 3486 Per- and Polyfluoroalkyl Substances (PFASs) from the OECD List. Environ. Sci. Technol. 2019, 53, 13970–13980. 10.1021/acs.est.9b04833. 31661253

[ref11] Wang Y.; Suzek T.; Zhang J.; Wang J.; He S.; Cheng T.; Shoemaker B. A.; Gindulyte A.; Bryant S. H. PubChem BioAssay: 2014 Update. Nucleic Acids Res. 2014, 42, D1075–D1082. 10.1093/nar/gkt978. 24198245 PMC3965008

[ref12] Rohrer S. G.; Baumann K. Maximum Unbiased Validation (MUV) Data Sets for Virtual Screening Based on PubChem Bioactivity Data. J. Chem. Inf. Model. 2009, 49, 169–184. 10.1021/ci8002649. 19434821

[ref13] Krewski D.; Acosta D.; Andersen M.; Anderson H.; Bailar J. C.; Boekelheide K.; Brent R.; Charnley G.; Cheung V. G.; Green S.; Kelsey K. T.; Kerkvliet N. I.; Li A. A.; McCray L.; Meyer O.; Patterson R. D.; Pennie W.; Scala R. A.; Solomon G. M.; Stephens M.; Yager J.; Zeise L.; Toxicity Testing in the 21st Century: A Vision and a Strategy. J. Toxicol. Environ. Health. B. Crit. Rev. 2010, 13, 51–138. 10.1080/10937404.2010.483176. 20574894 PMC4410863

[ref14] Subramanian G.; Ramsundar B.; Pande V.; Denny R. A. Computational Modeling of β-Secretase 1 (BACE-1) Inhibitors Using Ligand Based Approaches. J. Chem. Inf. Model. 2016, 56, 1936–1949. 10.1021/acs.jcim.6b00290. 27689393

[ref15] Martins I. F.; Teixeira A. L.; Pinheiro L.; Falcao A. O. A Bayesian Approach to in Silico Blood-Brain Barrier Penetration Modeling. J. Chem. Inf. Model. 2012, 52, 1686–1697. 10.1021/ci300124c. 22612593

[ref16] Rogers D.; Hahn M. Extended-Connectivity Fingerprints. J. Chem. Inf. Model. 2010, 50, 742–754. 10.1021/ci100050t. 20426451

[ref17] RDKit. http://www.rdkit.org/ (accessed June 29, 2021).

[ref18] Rousseeuw P. J. Silhouettes: A Graphical Aid to the Interpretation and Validation of Cluster Analysis. J. Comput. Appl. Math. 1987, 20, 53–65. 10.1016/0377-0427(87)90125-7.

[ref19] Morris G. M.; Huey R.; Lindstrom W.; Sanner M. F.; Belew R. K.; Goodsell D. S.; Olson A. J. Software News and Updates AutoDock4 and AutoDockTools4: Automated Docking with Selective Receptor Flexibility. J. Comput. Chem. 2009, 30, 2785–2791. 10.1002/jcc.21256. 19399780 PMC2760638

[ref20] Raies A. B.; Bajic V. B. In Silico Toxicology: Computational Methods for the Prediction of Chemical Toxicity. Wiley Interdiscip. Rev. Comput. Mol. Sci. 2016, 6, 147 10.1002/wcms.1240. 27066112 PMC4785608

[ref21] Yang H.; Lou C.; Li W.; Liu G.; Tang Y. Computational Approaches to Identify Structural Alerts and Their Applications in Environmental Toxicology and Drug Discovery. Chem. Res. Toxicol. 2020, 33, 1312–1322. 10.1021/acs.chemrestox.0c00006. 32091207

[ref22] Davies M.; Nowotka M.; Papadatos G.; Dedman N.; Gaulton A.; Atkinson F.; Bellis L.; Overington J. P. ChEMBL Web Services: Streamlining Access to Drug Discovery Data and Utilities. Nucleic Acids Res. 2015, 43, W612–W620. 10.1093/nar/gkv352. 25883136 PMC4489243

[ref23] Cheng X.; Klaassen C. D. Perfluorocarboxylic Acids Induce Cytochrome P450 Enzymes in Mouse Liver through Activation of PPAR-α and CAR Transcription Factors. Toxicol. Sci. 2008, 106, 29–36. 10.1093/toxsci/kfn147. 18648086 PMC2563145

[ref24] Miners J. O.; Birkett D. J. Cytochrome P4502C9: An Enzyme of Major Importance in Human Drug Metabolism. Br. J. Clin. Pharmacol. 1998, 45, 525–538. 10.1046/j.1365-2125.1998.00721.x. 9663807 PMC1873650

[ref25] Ashburner J.; Klöppel S. Multivariate Models of Inter-Subject Anatomical Variability. Neuroimage 2011, 56, 422–439. 10.1016/j.neuroimage.2010.03.059. 20347998 PMC3084454

[ref26] Chu C.; Hsu A. L.; Chou K. H.; Bandettini P.; Lin C. P. Does Feature Selection Improve Classification Accuracy? Impact of Sample Size and Feature Selection on Classification Using Anatomical Magnetic Resonance Images. Neuroimage 2012, 60, 59–70. 10.1016/j.neuroimage.2011.11.066. 22166797

[ref27] OECD Portal on Per and Poly Fluorinated Chemicals - OECD Portal on Per and Poly Fluorinated Chemicals. https://www.oecd.org/chemicalsafety/portal-perfluorinated-chemicals/ (accessed July 1, 2021).

