# Supplementary material for: Harnessing Semi-Supervised Machine Learning to Automatically Predict Bioactivities of Per- and Polyfluoroalkyl Substances (PFASs)
Source: Environ Sci Technol Lett. 2022 Aug 26;10(11):1017–22. doi: 10.1021/acs.estlett.2c00530 (PMC10653214; doi:10.1021/acs.estlett.2c00530)
Supplement: Supplementary file 1 — ez2c00530_si_001.pdf [file ez2c00530_si_001.pdf]

Supporting Information

Harnessing Semi-Supervised Machine Learning to Automatically Predict Bioactivities of Per- and Polyfluoroalkyl Substances (PFASs)

*Hyuna Kwon,[1] Zulfikhar A. Ali,[2] and Bryan M. Wong[1,2,]**

[1]Department of Chemical & Environmental Engineering and [2]Department of Physics & Astronomy, University of California-Riverside, Riverside, CA 92521, United States

*Corresponding author. E-mail: bryan.wong@ucr.edu. Website: http://www.bmwong-group.com

Contents

p. S3: Data Collection

p. S3, Table S1: Selected targets (ion channels, enzymes, and receptors)

p. S4: Discussion of Unsupervised Machine Learning Methods

p. S5: Model Selection

p. S6: Brief Discussion of Semi-Supervised Metric Learning

p. S7: Molecular Docking Calculations

p. S8, Table S2: Optimized hyperparameters and Silhouette scores for various unsupervised machine learning models

p. S9: Unsupervised Machine Learning Results

p. S10, Figure S1: Clustering of molecules in the C3F6 dataset

p. S11, Table S3: Cluster number, accuracy, and maximum common structure most likely to be found in bioactive molecules toward each target

p. S13, Figure S2: Clustering of molecules in the CF dataset

p. S14, Table S4: Predicted substructures that induce bioactivity toward each target, based on semi-supervised metric learning

p. S15, Figure S3: Representative molecules found in the CheMBL database (CHEMBL1411743, CHEMBL1411201, and CHEMBL1332759) that are bioactive on CYP2C9

p. S16, Figure S4: Histogram of binding energies between molecules in the CF dataset and CYP2C9

p. S17, Figure S5: Interactions between CYP2C9 and an ester-group-containing molecule (methyl 4-[2-propyl-1-({[4-trifluoromethyl)phenyl]sulfonyl}amino)-2-hexen-1-yl]benzoate), as predicted with Autodock

p. S18, Figure S6: Interactions between CYP and (a) ethyl-{[(nonafluorobutyl)sulfinyl]imino}acetate, (b) nonafluorohexyl2-chloroacrylate, (c) heptafluoropentyl acetate, and (d) hexafluorooxane-2,6-dione from the OECD list, as predicted with Autodock

p. S19: References

Data Collection

Our selection of proteins focuses on targets described previously in Cheng et al.,[1] and we screened the targets based on the availability of X-ray structures for our docking simulations (see Table S1).

Table S1: Selected targets (ion channels, enzymes, and receptors)

Target	PubChem AID	Description	PDB structure
CYP2C9	PCBA-883	Cytochrome p450 enzyme	1OG5
CYP3A4	PCBA-884	Cytochrome p450 enzyme	4D7D
CYP2D6	PCBA-891	Cytochrome p450 enzyme	5TFT
ATXN	PCBA-651635	Ataxin-2 protein	3KTR
K18	PCBA-1468	Protein-protein interaction	5HJC
VP16	PCBA-2546	Transcription factor	6T4X
ROR gamma	PCBA-2551	Nuclear receptor ROR gamma	6T4X

The bioactivity data from bioassays against 26 targets were extracted from the Supplementary Information of ref 1.

Discussion of Unsupervised Learning Methods

For the first unsupervised learning step, we used *dimension reduction* methods to project the high-dimensional fingerprint input data onto a 2-dimensional space. Two different dimension reduction methods, (i) Principal Component (PC) Analysis (PCA) followed by t-Distributed Stochastic Neighbor Embedding (t-SNE), i.e., PC t-SNE, and (ii) UMAP (Uniform Manifold Approximation and Projection for Dimension Reduction),[2] were used on our fingerprint data. While t-SNE is a widely-used dimension reduction technique for many types of data analysis, prior studies have shown that UMAP exhibits better clustering performance, especially for large datasets.[2] Thus, we decided to compare the clustering performance of both techniques.

After the dimension reduction procedure, we used the scikit-learn[3] library to execute three different *clustering methods*: k-means, Density-Based Spatial Clustering of Applications with Noise (DBSCAN), and Hierarchical DBSCAN (HDBSCAN). While k-means clustering is relatively more time-efficient, DBSCAN and HDBSCAN can efficiently handle outliers and noisy datasets. Since each method has clear advantages, we used all three techniques and evaluated their performances. By combining dimension reduction and clustering methods, we classified/grouped various PFAS structures based on their similarities.

Two different dimension reduction methods, PCA followed by t-SNE and UMAP[2] were used on the fingerprint data we generated. Three different clustering methods – k-means, DBSCAN, and HDBSCAN – were used after dimension reduction. Our work utilized the PCA/PC, t-SNE, k-means, DBSCAN, and HDBSCAN algorithms in the scikit-learn[3] library.

Model Selection

The final models were selected based on the best Silhouette score, which analyzes the distances of each data point to its cluster and neighboring clusters. In short, a higher Silhouette score guarantees better performance in clustering. The Silhouette score $s(i)$ of a data x_i can be calculated by the following expression:

$$s(i) = \frac{b(i) - w(i)}{\max\{b(i), w(i)\}} \text{ with } b(i) = \min_k\{B(i,k)\},$$

where $w(i)$ is the average distance from the ith point to the other points in its cluster, and $B(i,k)$ is the average distance from the ith point to points in another cluster k. The Silhouette score was also used as an essential statistical method to optimize hyperparameters.[4]

Brief Discussion of Semi-Supervised Metric Learning

Deep metric learning (DML) is a widely used machine learning technique to investigate mixed data distributions before building prediction models. DML approaches have been accurately used in state-of-the-art computer vision technologies,[5–7] and recently, Na et al. reported their usefulness in cheminformatics.[8] The central concept of DML is to make a prediction problem easy by creating a new vector representation and separating the data well depending on their target values (cf. Figure 2 in the main text).

The metric-learning algorithms compute/learn Mahalanobis distances, which are given by the expression:

$$D(x, x') = \sqrt{(Lx - Lx')^{\top}(Lx - Lx')}.$$

Based on the semi-supervised data, the metric learning problem is generally formulated as an optimization of Mahalanobis distances. That is, the metric-learning algorithm seeks to find the parameters of a distance function that optimizes some objective function measuring the agreement with the training data. In other words, we used metric-learning with a semi-supervised learning algorithm to learn a distance metric that places molecules with similar bioactivities close together and molecules with opposite bioactivities far away.

Molecular Docking Calculations

After identifying certain substructures that play a critical role in determining bioactivities, we simulated their interaction in the binding pocket of the targets to further probe the fundamental interactions giving rise to their enhanced bioactivity. To this end, AutoDock4[9–11] and Autodock Vina (v1.5.6)[12] were used to carry out the molecular docking procedure and visualize the docked conformations of the ligand-protein complexes.

The structures of the proteins for the docking analysis were obtained from the RCSB PDB database (htts://www.rcsb.org/). The other three-dimensional structures were constructed from SMILES using Open Babel (v2.4.0).[13] Autodock4 was used to process the ligand and ligand interaction conformation analysis in the binding affinity analysis. The docking pockets were based on the global search of the protein to determine the optimal binding sites.[10]

AutoDock4 uses a semiempirical free-energy forcefield scoring function to perform a quick and accurate evaluation of the binding energies of ligands to proteins using a two-step approach. First, the intramolecular energetics of the transition from the unbound state to the bound form of the protein-ligand complex is estimated, and the intermolecular energetics of the bound complex are subsequently evaluated.

Table S2: Optimized hyperparameters and Silhouette scores for various unsupervised machine learning models.

Dimension Reduction		Clustering			Silhouette Score
Model	Hyperparameters	Model	Hyperparameters	Number of clusters	
**PC t-SNE**	n_components = 60<br>perplexity = 40<br>n_iteration = 5000	**k-means**	n_clusters = 70	70	0.469
	n_components = 110<br>perplexity = 30<br>n_iteration = 1000	**DBSCAN**	eps = 0.07<br>min_samples = 3	137	0.379
	n_components = 50<br>perplexity = 30<br>n_iteration = 1000	**HDBSCAN**	eps = 0.09<br>min_samples = 3	63	0.414
**UMAP**	n_neighbor = 20	**k-means**	n_clusters = 10	10	0.577
	n_neighbor = 30	**DBSCAN**	eps = 0.03<br>min_samples = 3	77	0.456
	n_neighbor = 30	**HDBSCAN**	eps = 0.07<br>min_samples = 3	59	0.469

Unsupervised Machine Learning Results

In this section, we compare the performance of each machine learning algorithm in classifying/clustering the PFAS molecules based on their bioactivity (see Table S2 for their parameters and settings.) The parameters for each model are optimized for the best performance (i.e., the highest Silhouette score). As mentioned in the methods section, we tested six unsupervised learning models by combining two different dimension reduction methods (PC t-SNE and UMAP) with three different clustering methods (k-means, DBSCAN, and HDBSCAN). Table S2 shows that the combination of k-means clustering and the UMAP model exhibited better performance (with a Silhouette score of 0.577) than the other machine-learning methods.

Figure S1 displays the distribution based on the molecular structures of C3F6 datasets. First, the molecular structures were converted into constant-length arrays by ECFP. Next, the vector representations were projected onto a 2-dimensional space using the PC t-SNE dimension reduction methods (molecules that are grouped closer together indicate structural similarity). These molecules were subsequently clustered into ten groups using k-means clustering. We then used the bioactivity data from each target to analyze the molecules with respect to promising substructures leading to bioactivity.

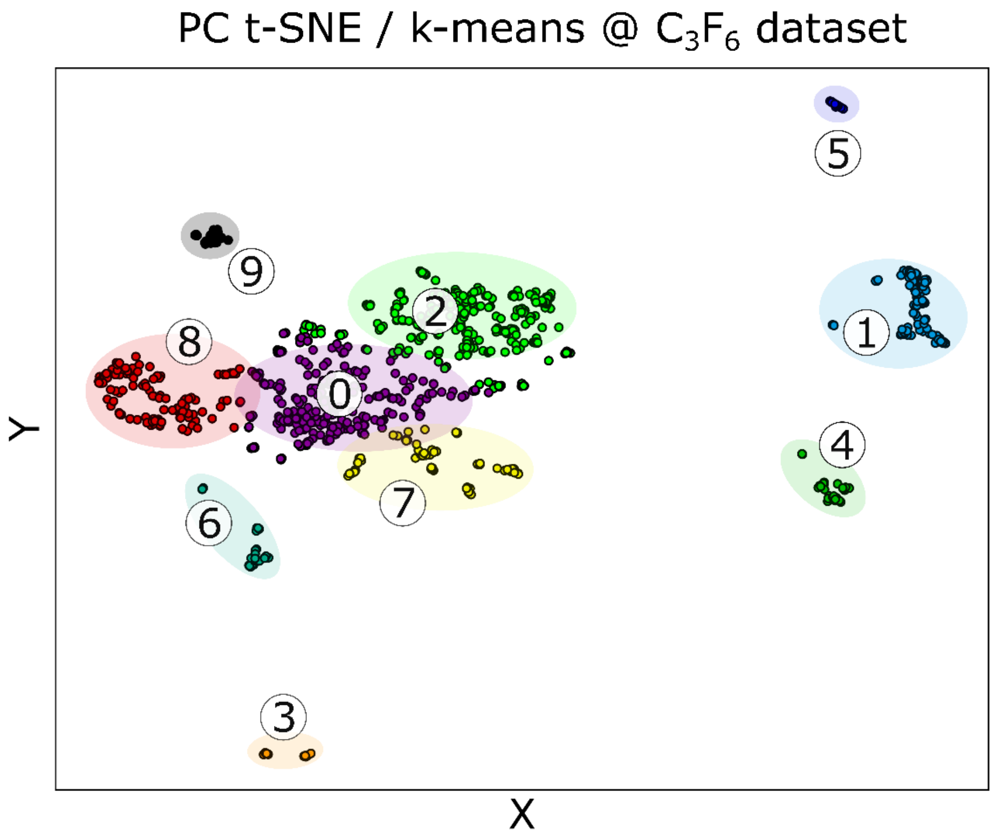

Figure S1: Clustering of molecules in the C3F6 dataset. PFAS molecules are represented on a 2-dimensional latent space using the PC t-SNE algorithm. Each point represents a molecule, and the colors of the points designate clusters classified using k-means clustering.

We found that 90.6%, 76.9%, and 70.0% of molecules in cluster 1 are bioactive on CYP2C9, CYP2D6, and CYP3A4, respectively. CYP2C9, CYP2D6, and CYP3A4 are members of Cytochrome p450 enzymes (Cyps) involved in metabolism processes by oxidizing xenobiotics in the human body. Cyps are major phase-I xenobiotic-metabolizing enzymes induced by many environmental xenobiotics and drugs.[14] Also, 42.9% of molecules in cluster 4 are bioactive on ATXN, a DNA-binding protein.[15,16] We only show targets with the top 4 true-positive rates of 90.6%, 76.9%, 70.0%, and 42.9%. The true positive rate is the probability that an actual positive (bioactive molecule) will be predicted positive (bioactive). Table S3 shows the maximum common substructures of the clusters.

Table S3: Cluster number, accuracy, and maximum common structure most likely to be found in bioactive molecules toward each target. Most molecules with 1,3-bis(trifluoromethyl) benzene are bioactive toward the CYP enzymes, while molecules with 1-ethyl-3,5-bis(trifluoromethyl) benzene are likely to be bioactive toward ATXN.

Target	Cluster	True-Positive Rate	Maximum Common Substructure
CYP2C9	1	90.6%	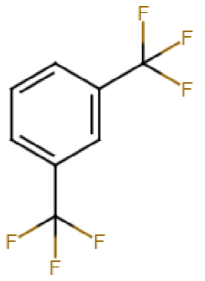 1,3-bis(trifluoromethyl) benzene
CYP2D6	1	76.9%	
CYP3A4	1	70.0%	
ATXN	4	42.9%	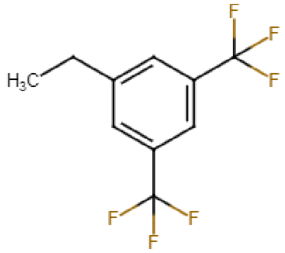 1-ethyl-3,5-bis(trifluoromethyl) benzene

Even though our unsupervised learning approaches could automatically classify PFASs into a reasonable number of clusters, it is essential to note that not all 26 targets clustered well (i.e., only ATXN, CYP2C9, CYP3A4, and CYP2D6 showed reasonable accuracy). From this result, we inferred that the bioactivity of PFAS against ATXN and Cyps have the strongest correlation with their molecular structures.

Furthermore, the larger CF dataset (containing 62,043 molecules) did not have a higher clustering performance than the smaller C3F6 dataset (containing 1,012 molecules) since traditional unsupervised learning clustering methods, such as k-means, work better when applied to smaller

datasets.[17] Because the CF dataset is 50 times larger than the C3F6 dataset and screened based on less rigorous criteria, we expect it to be more challenging to handle with unsupervised learning techniques.

For example, Figure SI-2a shows the bioactivities of molecules in the CF dataset toward keratin18 (K18), an intermediate filament protein.[18] Our unsupervised machine learning failed to successfully cluster the PFAS molecules based on their bioactivity toward K18, demonstrating a true-positive rate of only 55.9%. Similarly, Figure SI-2c shows the bioactivity toward CYP2C9 predicted with unsupervised learning, in which only 4.0% of the cluster showed bioactivity. These results highlight the inherent limitation of purely unsupervised learning for molecular structures. Since bioactivities are sensitive to minimal changes in molecular structures, they exhibit a mixed distribution in the molecular structure space. In other words, a pair of similar-structured molecules can have entirely different bioactivities, which are commonly referred to as activity cliffs in cheminformatics.[19–21]

To overcome the limitations of PC t-SNE and UMAP, we utilized semi-supervised metric learning to produce a more distinct separation between bioactive and inactive molecules toward K18, which can be seen in Figure SI-2b, which gives a true-positive rate of 79.2%. Similarly, Figure SI-2c shows the bioactivity toward CYP2C9 predicted by unsupervised learning in which, again, only 4.0% of the cluster showed bioactivity. Finally, Figure SI-2d shows the clustering based on semi-supervised metric learning, where 99.7% of a cluster's molecules are bioactive (i.e., a true positive rate of 99.7%).

It is not surprising that semi-supervised metric learning demonstrated significantly higher performance for PFAS QSAR classifications since it uses partially labeled data. In contrast, an unsupervised learning model takes unlabeled data as input. Furthermore, the performance difference is more significant with larger data, considering that traditional unsupervised learning methods, including k-means clustering, work better when applied to a smaller dataset.[17] Semi-supervised metric learning uses partially labeled bioactivity data as input data, placing molecules with similar bioactivities closer and opposite bioactivities further away. On the other hand, unsupervised learning tries to predict

bioactivities solely based on the chemical structure, making it extremely difficult to complete an appropriate QSAR task.

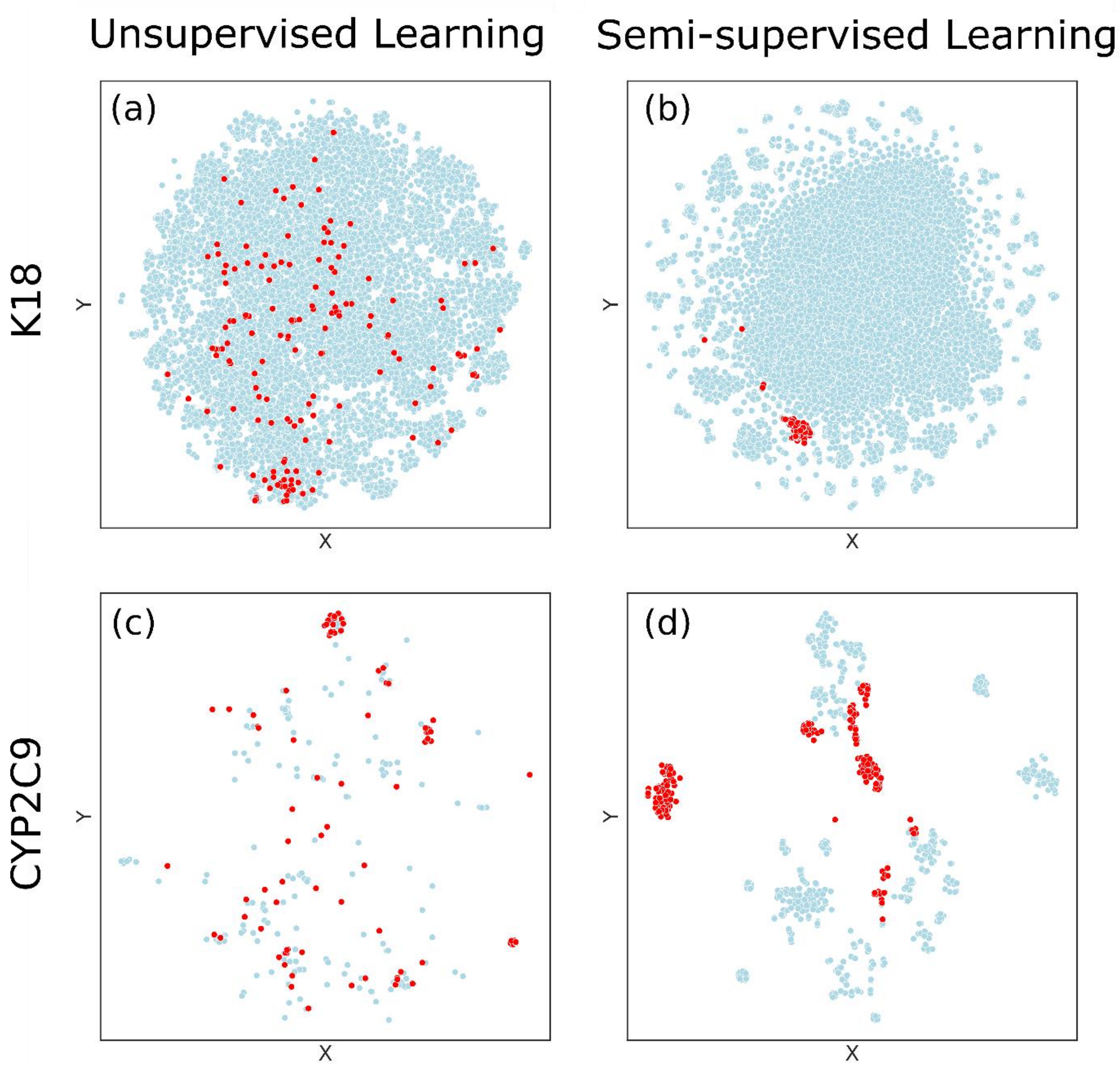

Figure S2: Clustering of molecules in the CF dataset. Each point represents a molecule that is either bioactive (red) or inactive (blue) towards (a, b) K18 and (c, d) CYP2C9. The molecules are visualized on a 2-dimensional space using (a, c) PC t-SNE (unsupervised) or (b, d) semi-supervised metric learning.

Table S4: Predicted substructures that induce bioactivity toward each target, based on semi-supervised metric learning.

Substructure	Target	Substructure	Target
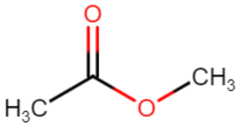 Ester	CYP2C9, CYP3A4	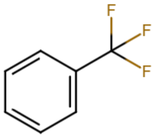 Trifluoromethylbenzene	CYP3A4, K18
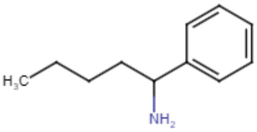 1-phenylpentan-1-amine	CYP2C9, CYP3A4	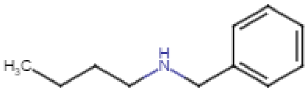 Benzyl(butyl)amine	CYP3A4
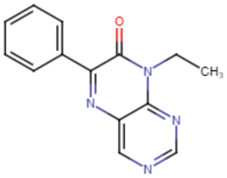 6-phenyl-8-ethyl-7(8H)-pteridinone	CYP2C9, CYP3A4	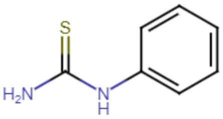 Phenylthiocarbamide	VP16, ROR Gamma
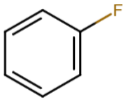 Fluorobenzene	CYP2C9, CYP3A4	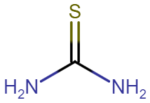 Thiocarbamide	ALDH1A1, VP16, ROR Gamma
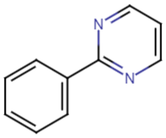 Phenylprimidine	CYP2D6	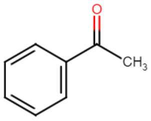 Phenylethanone	CYP2D6
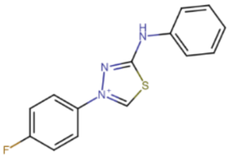 4-benzyl-2-(4-fluorophenyl)-1,2-thiazole	ATXN	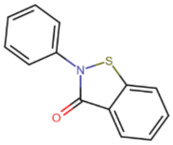 2-phenyl-2,3-dihydro-1,2-benzothiazol-3-one	ATXN

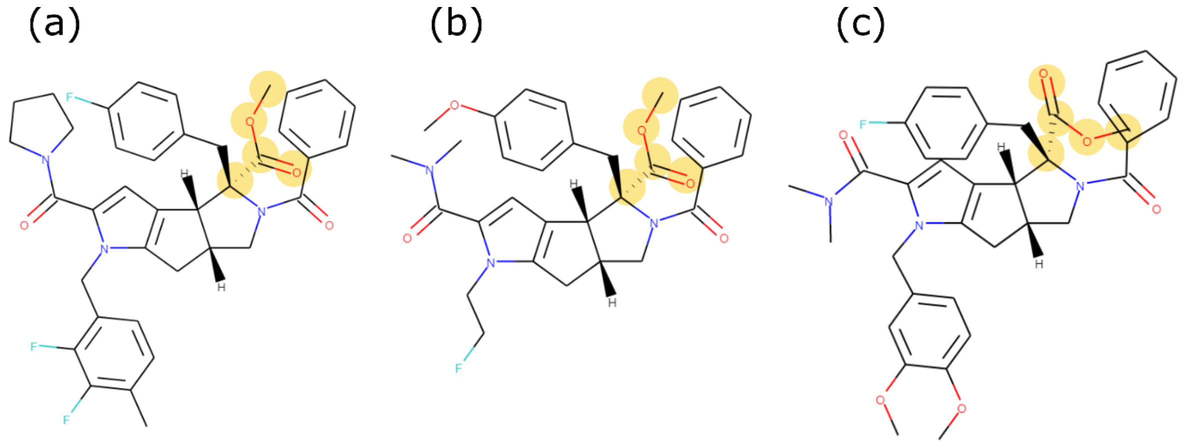

Figure S3: Representative molecules found in the CheMBL database that are bioactive on CYP2C9. The CheMBL IDs of the molecules are (a) CHEMBL1411743, (b) CHEMBL1411201, and (c) CHEMBL1332759. The yellow circles represent the functional groups resulting in a structural alert of each molecule.

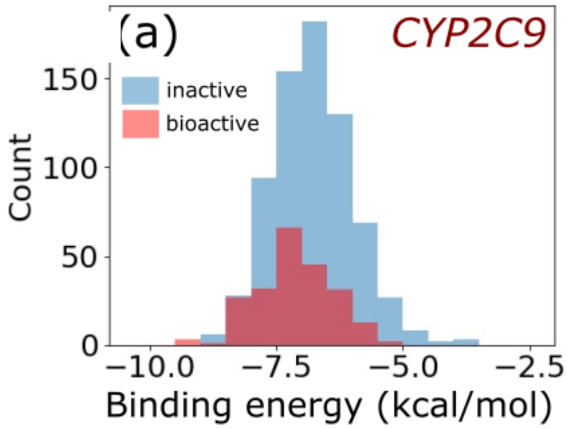

Figure S4: Histogram of binding energies between molecules in the CF dataset and CYP2C9. The histogram bins are organized by binding energies, where each bin represents the number of bioactive (red) and inactive (blue) molecules.

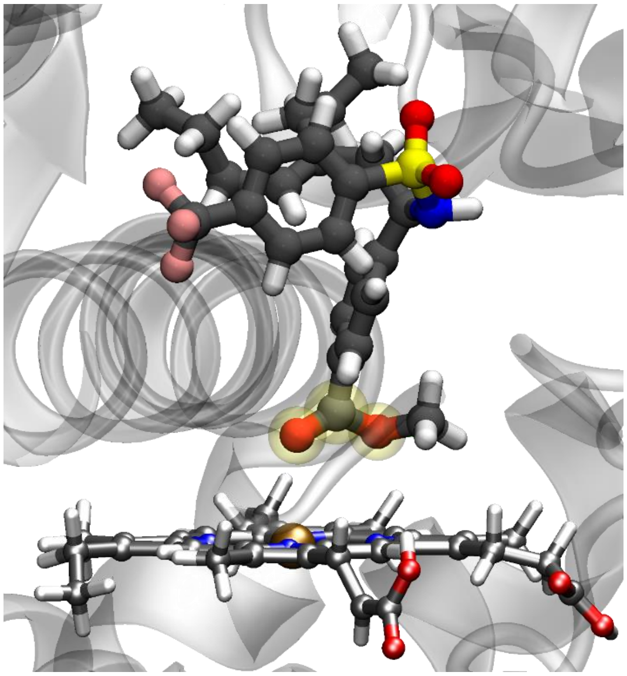

Figure S5: Interactions between CYP2C9 and an ester-group-containing molecule (methyl 4-[2-propyl-1-({[4-trifluoromethyl)phenyl]sulfonyl}amino)-2-hexen-1-yl]benzoate), as predicted with Autodock. The ester group is highlighted with yellow spheres. The planar structure at the bottom represents the HEME group in CYP2C9. The C, N, O, F, and Fe atoms are colored grey, blue, red, pink, and brown, respectively.

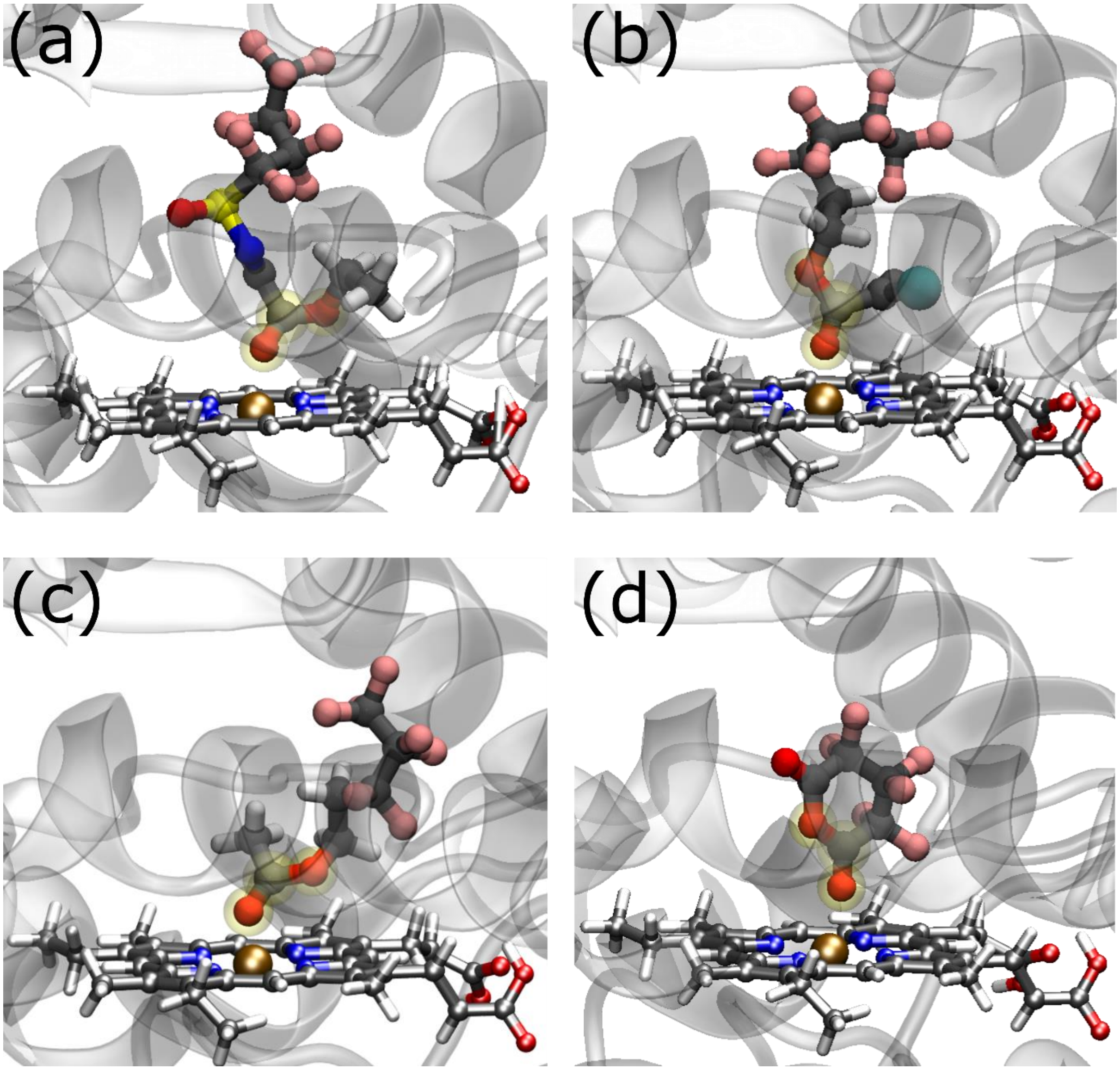

Figure S6: Interactions between CYP and ester-containing PFAS ligands, (a) ethyl-{[(nonafluorobutyl)sulfinyl]imino}acetate, (b) nonafluorohexyl2-chloroacrylate, (c) heptafluoropentyl acetate, and (d) hexafluorooxane-2,6-dione from the OECD list, as predicted with Autodock. The ester groups are highlighted with yellow spheres. The planar structure at the bottom represents the HEME group of CYP2C9. The C, N, O, F, and Fe atoms are colored grey, blue, red, pink, and brown, respectively.

References

(1) Cheng, W.; Ng, C. A. Using Machine Learning to Classify Bioactivity for 3486 Per- And Polyfluoroalkyl Substances (PFASs) from the OECD List. *Environ. Sci. Technol.* **2019**, *53*, 13970–13980.

(2) McInnes, L.; Healy, J.; Saul, N.; Großberger, L. UMAP: Uniform Manifold Approximation and Projection. *J. Open Source Softw.* **2018**, *3*, 861.

(3) Pedregosa, F.; Varoquaux, G.; Gramfort, A.; Michel, V.; Thirion, B.; Grisel, O.; Blondel, M.; Prettenhofer, P.; Weiss, R.; Dubourg, V.; Vanderplas, J.; Passos, A.; Cournapeau, D.; Brucher, M.; Perrot, M.; Duchesnay, E. Scikit-learn: Machine Learning in Python. *J. Mach. Learn. Res.* **2011**, *12*, 2825−2830.

(4) Shahapure, K. R.; Nicholas, C. Cluster Quality Analysis Using Silhouette Score. In *Proceedings - 2020 IEEE 7th International Conference on Data Science and Advanced Analytics, DSAA 2020* **2020**, 747–748.

(5) Kaya, M.; Bilge, H. S. Deep Metric Learning : A Survey. *Symmetry (Basel)* **2019**, *11*, 1066.

(6) Sohn, K. Improved Deep Metric Learning with Multi-Class N-Pair Loss Objective. *Adv. Neural Inf. Process Syst.* **2016**, 1857–1865.

(7) Davis, J. v.; Kulis, B.; Jain, P.; Sra, S.; Dhillon, I. S. Information-Theoretic Metric Learning. *ACM International Conference Proceeding Series* **2007**, *227*, 209–216.

(8) Na, G. S.; Chang, H.; Kim, H. W. Machine-Guided Representation for Accurate Graph-Based Molecular Machine Learning. *Phys. Chem. Chem. Phys.* **2020**, *22*, 18526–18535.

(9) Morris, G. M.; Huey, R.; Lindstrom, W.; Sanner, M. F.; Belew, R. K.; Goodsell, D. S.; Olson, A. J. Software News and Updates AutoDock4 and AutoDockTools4: Automated Docking with Selective Receptor Flexibility. *J. Comput. Chem.* **2009**, *30*, 2785–2791.

(10) Morris, G. M.; Huey, R.; Olson, A. J. Using AutoDock for Ligand-Receptor Docking. *Current Protocols in Bioinform*. **2008**, 8.14.1-8.14.40.

(11) Salentin, S.; Schreiber, S.; Haupt, V. J.; Adasme, M. F.; Schroeder, M. PLIP: Fully Automated Protein-Ligand Interaction Profiler. *Nucleic Acids Res* **2015**, *43*, W443–W447.

(12) Trott, O.; Olson, A. J. AutoDock Vina: Improving the Speed and Accuracy of Docking with a New Scoring Function, Efficient Optimization and Multithreading. *J. Comput. Chem.* **2010**, *31*, 455.

(13) O'Boyle, N. M.; Banck, M.; James, C. A.; Morley, C.; Vandermeersch, T.; Hutchison, G. R. Open Babel: An Open Chemical Toolbox. *J. Cheminform.* **2011**, *3*, 1–14.

(14) Cheng, X.; Klaassen, C. D. Perfluorocarboxylic Acids Induce Cytochrome P450 Enzymes in Mouse Liver through Activation of PPAR-α and CAR Transcription Factors. *Toxicol. Sci.* **2008**, *106*, 29–36.

(15) Volz, A.; Fonatsch, C.; Ziegler, A. Regional Mapping of the Gene for Autosomal Dominant Spinocerebellar Ataxia (SCA1) by Localizing the Closely Linked D6S89 Locus to 6p24.2→p23.05. *Cytogenet Genome Res.* **1992**, *60*, 37–39.

(16) Leysen, S.; Burnley, R. J.; Rodriguez, E.; Milroy, L. G.; Soini, L.; Adamski, C. J.; Nitschke, L.; Davis, R.; Obsil, T.; Brunsveld, L.; Crabbe, T.; Zoghbi, H. Y.; Ottmann, C.; Davis, J. M. A Structural Study of the Cytoplasmic Chaperone Effect of 14-3-3 Proteins on Ataxin-1. *J. Mol. Biol.* **2021**, *433*.

(17) Sreedhar, C.; Kasiviswanath, N.; Chenna Reddy, P. Clustering Large Datasets Using K-Means Modified Inter and Intra Clustering (KM-I2C) in Hadoop. *J. Big Data* **2017**, *4*, 1–19.

(18) Schnabel, J.; Weber, K.; Hatzfeld, M. Protein–Protein Interactions between Keratin Polypeptides Expressed in the Yeast Two-Hybrid System. *Biochimica et Biophysica Acta (BBA) - Molecular Cell Res.* **1998**, *1403*, 158–168.

(19) Cruz-Monteagudo, M.; Medina-Franco, J. L.; Pérez-Castillo, Y.; Nicolotti, O.; Cordeiro, M. N. D. S.; Borges, F. Activity Cliffs in Drug Discovery: Dr Jekyll or Mr Hyde? *Drug Discov. Today* **2014**, *19*, 1069–1080.

(20) Stumpfe, D.; Hu, Y.; Dimova, D.; Bajorath, J. Recent Progress in Understanding Activity Cliffs and Their Utility in Medicinal Chemistry. *J. Med. Chem.* **2014**, *57*, 18–28.

(21) Hu, H.; Bajorath, J. Systematic Exploration of Activity Cliffs Containing Privileged Substructures. *Mol. Pharm.* **2020**, *17*, 979–989.
